# Clinical Examination of the Urine and Urinary Diagnosis

**Published:** 1900-12

**Authors:** 


Clinical Examination of the Urine and Urinary Diagnosis. A Clinical Guide for the Use of Practitioners and Students of Medicine and Surgery. By J. Bergen Ogden, M. D., Instructor in Chemistry, Harvard University Medical School; Assistant in Clinical Pathology, Boston City Hospital, etc. Octavo, pp. 416. Illustrated. Philadelphia: W. B. Saunders & Co. 1900. [Price, $3.00 net.]

The author's purpose in putting forth another work on the urine is to present in as concise a manner as possible the chemistry of the urine and its relation to physiologic processes; the most approved working methods, both qualitative and quantitative; the diagnosis of diseases and disturbances of the kidneys and urinary passages. It is saying very little, when it is said that the author has succeeded admirably and has given the profession an exposition of the subject, second to none in the English language. The work naturally falls into two parts. In part I. chemic and microscopic methods are described in detail, and numerous illustrations, many of which are original have been introduced, thus enabling the student and practitioner who has not had special training in urinary analysis to obtain accurate results. The normal and abnormal constituents of the urine are plainly set forth, and the causes given which lead to the formation of the various abnormal constituents. The chapter on urea is a very interesting and typical one; the author after giving the chemical properties of urea, follows up with the various tests for its detection, its quantity in health and disease, whether increased or diminished, and the causes leading up to its variableness. The other constituents are treated in the same thorough-going manner.

In part II. special attention has been paid to diagnosis, which includes our present knowledge of the character of the urine, the diagnosis and differential diagnosis of disturbances and diseases of the kidneys and urinary passages, whether they be local or general, medical or surgical, a brief enumeration of the prominent clinical symptoms of each disease, and, finally, the peculiarities of the urine in certain general diseases of the body.

Appendix *A*, which follows, gives the author's method of recording urinary examinations and a modified tabular arrangement of Heller's tests. The order of applying tests is then considered, and also the method of making diagnoses of diseases of the kidneys from the urine. These directions are very important, especially to the large body of physicians who have not worked sufficiently in the chemico-urinary laboratories, and add greatly to the usefulness and practicability of the volume.

In appendix *B*, is given a list of the reagents and apparatus necessary for the qualitative and quantitative analysis of the urine.

The illustrations and colored plates are handsomely executed and are profusely scattered throughout the book. The binding is in good taste, and the volume is a credit both to author and publisher.

